# Memory deficiency, cerebral amyloid angiopathy, and amyloid-β plaques in APP+PS1 double transgenic rat model of Alzheimer’s disease

**DOI:** 10.1371/journal.pone.0195469

**Published:** 2018-04-11

**Authors:** Diana Klakotskaia, Cansu Agca, Rachel A. Richardson, Edward G. Stopa, Todd R. Schachtman, Yuksel Agca

**Affiliations:** 1 Department of Psychological Sciences, University of Missouri, Columbia, Missouri, United States of America; 2 Department of Veterinary Pathobiology, College of Veterinary Medicine, University of Missouri, Columbia, Missouri, United States of America; 3 Department of Pathology, Rhode Island Hospital, Providence, Rhode Island, United States of America; Nathan S Kline Institute, UNITED STATES

## Abstract

Transgenic rat models of Alzheimer’s disease were used to examine differences in memory and brain histology. Double transgenic female rats (APP+PS1) over-expressing human amyloid precursor protein (APP) and presenilin 1 (PS1) and single transgenic rats (APP21) over-expressing human APP were compared with wild type Fischer rats (WT). The Barnes maze assessed learning and memory and showed that both APP21 and APP+PS1 rats made significantly more errors than the WT rats during the acquisition phase, signifying slower learning. Additionally, the APP+PS1 rats made significantly more errors following a retention interval, indicating impaired memory compared to both the APP21 and WT rats. Immunohistochemistry using an antibody against amyloid-β (Aβ) showed extensive and mostly diffuse Aβ plaques in the hippocampus and dense plaques that contained tau in the cortex of the brains of the APP+PS1 rats. Furthermore, the APP+PS1 rats also showed vascular changes, including cerebral amyloid angiopathy with extensive Aβ deposits in cortical and leptomeningeal blood vessel walls and venous collagenosis. In addition to the Aβ accumulation observed in arterial, venous, and capillary walls, APP+PS1 rats also displayed enlarged blood vessels and perivascular space. Overall, the brain histopathology and behavioral assessment showed that the APP+PS1 rats demonstrated behavioral characteristics and vascular changes similar to those commonly observed in patients with Alzheimer’s disease.

## Introduction

Alzheimer’s disease (AD) is one of the most devastating and costly diseases in the world and currently affects over 5.5 million Americans [1]. One of the defining physiological characteristics of the disease is the progressive accumulation of amyloid-β (Aβ) plaques in the brain, which results from the aggregation of Aβ peptides. The two major forms of Aβ peptides, Aβ40 and Aβ42, are produced by the cleavage of amyloid precursor protein (APP) by ϒ-secretase, which is a protease complex of four proteins that includes presenilin 1 (PS1). Mutations in the PS1 gene result in early-onset AD and lead to the overproduction of Aβ42 which is prone to forming aggregates and plaques [2]. As a consequence of Aβ accumulation, vascular changes such as cerebral amyloid angiopathy (CAA), characterized by Aβ deposition in leptomeningeal and cortical vessel walls [3, 4], and Aβ-related angiitis, characterized by immune activation around vessels, are also commonly observed in AD. Pathologic changes in AD patients are mainly observed in the brain cortex and hippocampus. Subregions of the hippocampus show differential gene expression which corresponds to differential emotional and cognitive functions [5]. Furthermore, subregions of the hippocampus, dentate gyrus (DG), and Cornu Ammonis (CA1 and CA3) are associated with various behavioral functions. CA1 plays a role in temporal pattern association and completion in addition to intermediate term memory and CA2 plays a role in spatial pattern association and completion as well as short term memory [6].

Experimental animals provide a model for investigating the role of gene mutations in AD. Transgenic animal models of AD mimic the disease by overexpressing APP and PS1 genes that have been linked to the familial form of the disease. The overexpression of these genes in animals leads to various AD phenotypes. For instance, APP Dutch mice have been found to mainly show CAA, whereas APP Dutch mice that have been crossed with PS45 (PS1-overexpressing) mice show extensive plaques in the cortex and hippocampus [7]. Contrary to many mouse models of AD, APP21 rats that overexpress human APP using the Swedish and Indiana mutations do not produce Aβ plaques in their brains [8]. To induce plaques, the APP21 model was used to generate a double transgenic rat that overexpresses both human APP and PS1 (APP+PS1) [9]. Based on previous studies that have found greater behavioral impairment and pathology in females across multiple transgenic (Tg) mouse models and recognizing the notion that females may be inherently more vulnerable than males to AD pathogenesis [10], the focus of the current study was to characterize the behavioral symptoms and pathology of older female APP+PS1 rats.

In this experiment, female rats underwent behavioral assessment in the Barnes maze. The Barnes maze was chosen because it is a hippocampal-dependent task that been shown to be sensitive to both hippocampal damage and to cognitive decline in AD rodents. This maze is particularly well-suited to examining learning in animal models of AD because it does not rely on appetitive motivation and is considered to be less stressful than other common tasks like the Morris water maze [11]. Previous research found that Tg animals display poor spatial learning in the Barnes maze task compared to their wild-type littermates in a variety of AD mouse models, including TgCRND8 [12], 3xTg [13], APPswe/PS1ΔE9 [14,15] as well as the APP21 and APP+PS1 rats [9]. After behavioral testing, the brains from a subset of animals were analyzed for the presence of Aβ plaques and tau using immunohistochemistry. Subsequently, changes in the vasculature of the brain cortex and hippocampus were also evaluated.

## Methods

### Animals

The APP21 and APP+PS1 rats were littermates and were bred and born in the facility in which the experiments were performed. All of the Tg rats were homozygous for the human APP transgene. In addition to the APP transgene, the APP+PS1 rats were hemizygous for the human PS1 transgene. The wild type Fischer 344 (WT) rats were purchased from Envigo (Indianapolis, IN) at 4 weeks of age. Rats were housed in conventional cages at 20–25°C with free access to food and water and all of the animal studies performed were approved by the University of Missouri’s Animal Care and Use Committee and were in accordance with the guidelines of the Institute for Laboratory Animal Research Guide for the Care and Use of Laboratory Animals.

### Behavioral measures

Behavioral assessment was conducted using 12-mo and 14-mo APP+PS1 (*n* = 5; *n* = 7), APP21 (*n* = 10; *n* = 7), and WT (*n* = 10; *n* = 7) female rats. Prior to the start of behavioral testing, each rat was handled at least 4 times to acclimate them to being handled during behavioral testing. On each day that behavioral testing occurred, rats were transported to the testing room from the colony room in their home cages and acclimated in the testing room for a minimum of 30 min prior to the first trial.

#### Barnes maze

The maze consisted of a grey circular platform 122 cm in diameter, surrounded by a wall that was 30.5 cm in height. The maze was elevated 83.8 cm above the floor by a stand. Twenty holes measuring 10.2 cm in diameter were evenly spaced around the perimeter. A rectangular grey goal box (28 cm in length × 12.7 cm wide × 7.6 cm high at the area closest to the maze tapering to 16.5 cm high) could be placed beneath any hole. The goal box included an entry ramp that provided easy entry access for the rat. Black curtains were hung around the maze and above the maze walls to surround the apparatus and ensure that rats could only use the visual cues provided in the maze, rather than the distal cues within the testing room, to navigate around the maze. Proximal cues were more likely to remain constant, within subjects and across subjects, during the course of training. Four visual cues consisting of various shapes (triangle, square, circle, cross) were placed at evenly spaced intervals on the inside of the maze walls. Two 86-W, 120-V floodlights producing 1690 lumins were hung above the platform served to brightly light the maze in order to create a potentially aversive environment to help motivate the rats to escape from the brightly lit, open surface in favor of the dark environment of the goal box. One light hung 68.5 on side of the maze while the other hung 137 cm from the other side.

Each rat was assigned a goal box location; goal box location was alternated across rats to eliminate odor cues for consecutively tested rats. The goal box location remained constant for any individual rat across test trials. Before the start of behavioral testing, each rat was pre-exposed to the goal box for 90 s. Behavioral testing consisted of 9 acquisition trials (3 trials/day) over a period of 3 days.

An acquisition trial began by placing the rat under a grey, opaque start box (23 cm length x 23 cm wide) positioned in the center of the platform. After 30 s, the box was lifted and the rat had a maximum of 3 min (180 s) to locate and enter the goal box. Latency (time it took for the rat to find and enter the goal box) and total errors (nose-pokes into non-escape holes) were recorded. If the rat did not enter the goal box within 3 min, it was gently guided there by the experimenter’s hand. Once the rats had entered the goal box, the entrance of the goal box was covered with the start box to prevent escape back onto the maze platform. After 30 s, the rat was removed from the goal box and returned to its home cage. Rats were allowed to rest in their home cage in the testing room for 30 min before starting their subsequent daily trial. The platform and goal box were cleaned after every trial with a 20% ethanol solution. After the third day, testing abated for 14 days, after which retention was evaluated with 3 additional trials (1 day), conducted exactly like previous acquisition trials. The following day, rats were given reversal training, in which the rats were given trials exactly like those of original acquisition training, except that the goal box was moved to a new location in the opposite quadrant. Reversal training continued for a total of 3 days.

### Immunohistochemistry

Brains from 6 APP+PS1, 3 APP21, and 4 WT female rats at 19-mos of age were used for the histological analysis. The rats which were used in the analysis also underwent brain MRI under isoflurane anesthesia a week before brain collection. Brains were fixed for 48 hrs at room temperature in 10% buffered formalin and embedded in paraffin. Eight μm thick tissue sections were placed on poly-L-lysine-coated slides and incubated in at 60°C for 1 hr, and after deparaffinization and rehydration, they were treated with 10 mM citrate buffer (85°C; pH 6) for 15 min. To eliminate endogenous peroxidase activity, sections were washed with distilled water and then quenched with a dual endogenous enzyme-blocking reagent (Agilent, Santa Clara, CA, USA; Catalog # S200380-2) for 10 min at room temperature. Additionally, a supplementary 2-min pretreatment with 70% Formic acid at room temperature was performed for Aβ immunohistochemistry. The sections were incubated for 30 min at room temperature with rabbit polyclonal antibodies directed against tau (Agilent, Santa Clara, CA, USA; Catalog # A002401-2, diluted 1:600), or with mouse monoclonal antibodies directed against Aβ (6E10; Biolegend Inc., Catalog # 800709). After washing in 0.05 M Tris-buffered saline with 0.05% Tween-20 (TBST; pH 7.6), a horseradish peroxidase labeled polymer conjugated with secondary antibodies against rabbit and mouse antibodies (EnVision+ Dual Link Kit; Agilent Santa Clara, CA, USA; Catalog # K406511-2) was applied for 30 min at room temperature, in accordance with the EnVision + Dual Link Kit for immunohistochemical staining. The tissue sections were washed with TBST and 3,3-diaminobenzidine (Agilent Santa Clara, CA, USA; Catalog # K346811-2) was used as the chromogen to develop the immunoreaction. Sections were dehydrated and coverslips were sealed using Cytoseal XYL (Richard-Allan Scientific, Kalamazoo, MI, USA; Catalog #8312–4). Primary antibody omission controls were run with the samples to check for non-specific binding due to the secondary antibodies, and advanced AD human prefrontal cortical sections were used as positive controls.

### Masson trichrome staining

Brains from six animals from each APP+PS1, APP21, and WT groups were used in Masson trichrome staining using a protocol similar to the one described by Silverberg and colleagues [16]. Following deparaffinization, tissue sections were hydrated in distilled water and incubated in Bouin’s fixative (Richard-Allan Scientific; Catalog #NC9674780) for 1 hr at 56°C. Sections were stained in Weigert’s iron hematoxylin (Richard-Allan Scientific; Catalog #NC9231529) for 10 min and then in Biebrich scarlet-acid fuchsin (Richard-Allan Scientific; Catalog #NC9424144) for 2 min. Subsequently, sections were incubated in phosphomolybdic-phosphotungstic acid (Richard-Allan Scientific; Catalog #NC9443038), followed by aniline blue (Richard-Allan Scientific; Catalog #NC9684104) for 10 min each. The sections were differentiated in 1% acetic acid for 3 min and dehydrated before sealing using Cytoseal XYL.

### Verhoeff-Van Gieson staining

The Verhoeff-Van Gieson staining for elastic fibers was similar to the previously described protocol by Silverberg and colleagues [16]. Tissue sections were deparaffinized, hydrated, and incubated in Verhoeff’s working solution (Polysciences, Warrington, PA, USA; Catalog #25089) for 1 hr. Sections were then differentiated in 2% ferric chloride (Sigma-Aldrich; Catalog #451649) for 2 min and treated with 5% aqueous sodium thiosulfate (Sigma-Aldrich; Catalog #S7026) for 1 min. Van Gieson’s solution (Poly Scientific, Bay Shore, NY, USA; Catalog #s289) was used for counterstaining and was followed by dehydration and sealing using Cytoseal XYL.

### Microscopy, image acquisition & qualitative grading

The histology slides were analyzed using a Nikon Eclipse E600 microscope (Melville, NY) and an Olympus DP72 camera (Center Valley, PA). Small magnification pictures were taken using a Leica DM IL LED microscope (Buffalo Grove, IL). Image J Fiji version [17] was used to analyze Aβ area in the hippocampus, blood vessels, and perivascular area, as well as, the number of necrotic cells in the frontal cortex.

### Amyloid-β 40 and 42 ELISA

Blood samples were collected from the same rats that were used for the histological analysis. Blood was obtained from 6 APP+PS1 and 5 APP21 rats postmortem by cardiac puncture and serum was separated and frozen at -80°C. Serum from rats was analyzed for Aβ40 and Aβ42 using Invitrogen’s (Carlsbad, CA) human Aβ40 (catalog number KHB3481) and Aβ42 (catalog number KHB3441) kits according the manufacturer’s protocol. Serum samples were diluted in standard diluent buffer and 1ul protease inhibitor cocktail (Sigma catalog number P8340) was added and transferred to Aβ antibody coated ELISA plates. A human Aβ40 or Aβ42 detection antibody was added to each well and incubated at room temperature for 3 hrs. Following incubation, wells were washed and incubated with antirabbit IgG HRP followed by chromogen solutions for 30 min each. The reactions were stopped and absorbance was determined at 450 nm and Aβ concentration in the samples was calculated from standard curves.

### Statistical analysis

Behavioral analyses were conducted using SPSS (SPSS 21, SPSS Inc., Chicago, USA) and analysis of variance (ANOVA). For the Barnes maze, daily latency and error performance was averaged each day and used in the repeated-measures analysis. *Day* was used as the within-subjects factor and *genotype* and *age* were used as between-group factors. Comparisons of performance were analyzed separately for each training phase. General linear models (GLM) procedure of SAS (version 9.3; Cary, NC) was used to analyze areas of diffuse and dense Aβ plaques, blood vessels, collagenous blood vessels and perivascular area, as well as necrotic cells. The proc GLM model was area (of plaque or vessel) = genotype. Differences were considered significant at *P*<0.05 for all analyses and multiple comparisons were conducted using Duncan’s multiple range test.

## Results

### Behavioral changes

#### Acquisition

**Fig 1:**
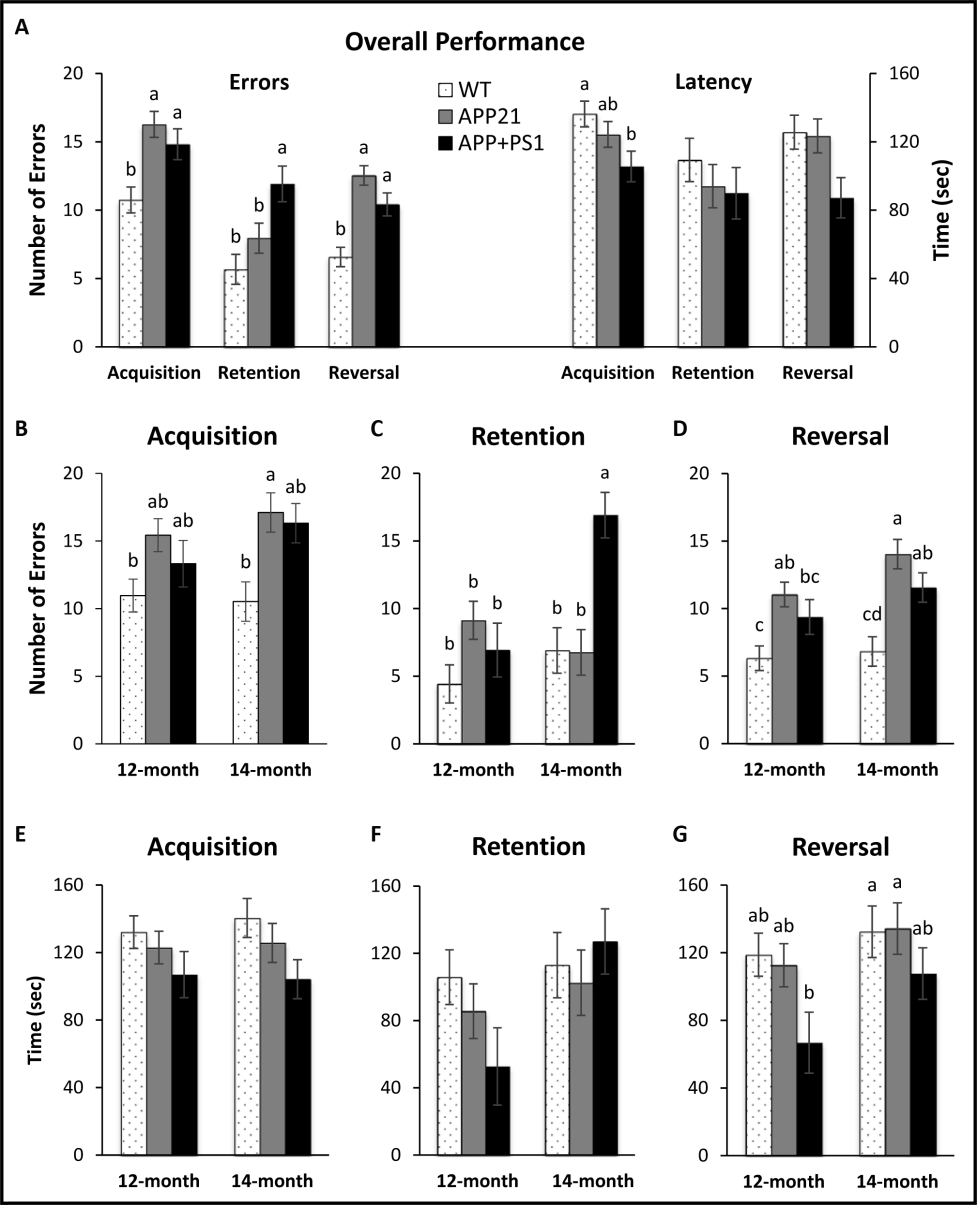
Barnes maze results. (A) Overall error and latency performance by genotype collapsed over age and training days for each phase; (B) Acquisition training errors by age; (C) Retention training errors by age; (D) Reversal training errors by age; (E) Acquisition training latency by age; (F) Retention training latency by age; (G) Reversal training latency by age. Letters indicate significant differences between groups (*P*<0.05).

Overall, both error and latency performance significantly improved across acquisition training days (*F*s_(2,80)_>7.32, *P*s<0.002; data not shown). There was a significant main effect of genotype for errors (*F*_(2,40)_ = 8.98, *P* = 0.001), indicating WT animals made significantly fewer errors than APP21 (*P* = 0.002) and APP+PS1 animals (*P* = 0.02), as shown in Fig 1A. Additionally, there was a significant main effect of genotype for latency (*F*_(2,40)_ = 3.45, *P* = 0.04), showing WT animals had unexpectedly significantly longer latencies than APP+PS1 animals (*P* = 0.04; Fig 1A). Although there were no significant differences in latency or error performance between 12-mo and 14-mo rats when collapsed over genotype (*Fs*_(1,40)_<1.46, *Ps*>0.23; Fig 1B shows acquisition error performance for all 6 groups and Fig 1E shows acquisition latency performance for all 6 groups), there was a significant three-way interaction for errors (*F*_(4,80)_ = 2.57, *P*<0.05), and 14-mo old APP21 rats showed a steep improvement in error performance on Day 3, whereas 12-mo old APP21 rats did not (data not shown).

#### Retention

Error performance during the retention test showed significant main effects of genotype (*F*_(2,40)_ = 6.75, *P*<0.01), age (*F*_(1,40)_ = 6.18, *P* = 0.02), and a genotype by age interaction (*F*_(2,40)_ = 6.56, *P*<0.01). Latency performance during the retention test showed only a significant main effect of age (*F*_(1,40)_ = 4.42, *P* = 0.04). More specifically, APP+PS1 rats made significantly more errors than both APP21 (*P* = 0.03) and WT animals (*P* = 0.001) when collapsed over age as shown in Fig 1A. Additionally, when collapsed over genotype, 14-mo old rats made significantly more errors (*P* = 0.02; Fig 1C shows retention error performance for all 6 groups) and had significantly longer latencies than 12-mo old rats (*P*<0.05; Fig 1F shows retention latency performance for all 6 groups). In both instances, these differences were driven largely by poor performance in the 14-mo old APP+PS1 rats.

#### Reversal

There was a significant main effect of genotype for errors (*F*_(2,40)_ = 18.04, *P<*0.001), indicating WT animals made significantly fewer errors than both APP21 (*P* = 0.001) and APP+PS1 animals (*P* = 0.003) as shown in Fig 1A. Although there was a significant main effect of genotype for latencies (*F*_(2,40)_ = 3.70, *P*<0.04), post-hoc tests found no significant genotype differences in latencies and showed that APP+PS1 rats had a non-significant tendency to have shorter latencies than both the WT (*P* = 0.09) and the APP21 rats (*P* = 0.12). Additionally, as expected, when collapsed over genotype, 14-mo old animals made significantly more errors and had significantly longer latencies than 12-mo old rats (*F*s_(1,40)_>4.30, *P*s<0.05; Fig 1D shows reversal error performance for all 6 groups and Fig 1G shows reversal latency performance for all 6 groups). More specifically, for errors, this difference was driven by poor performance in the 14-mo old APP21 and APP+PS1 rat counterparts, while for latency, this difference was due to poor performance in all of the 14-mo old genotype groups compared with 12-mo old genotype groups.

### Amyloid-β deposition and plaque formation

**Fig 2:**
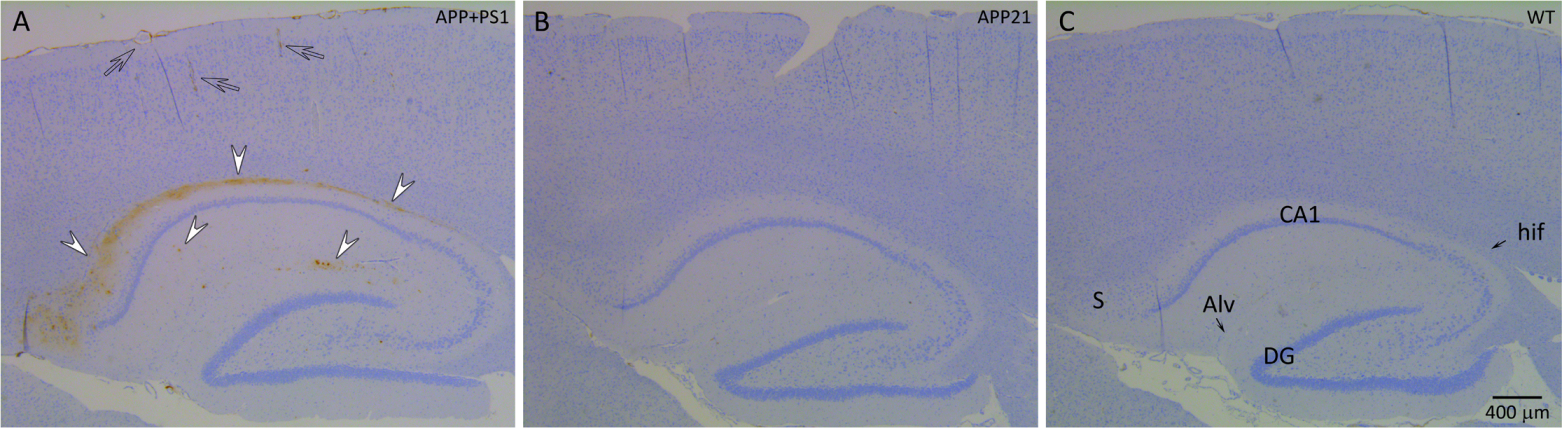
**Immunohistochemistry of the hippocampus and the cortex of an APP+PS1 (A), APP21 (B) and WT (C) rat using 6E10 monoclonal antibody raised against Aβ.** Brown staining indicates the presence of Aβ. A. APP+PS1 rat with extensive, mostly diffuse Aβ plaques (arrow heads) under alveolus hippocampus (Alv), above CA1 pyramidal layer of hippocampus, as well as subiculum (S). In addition, several dense plaques (arrow heads) were present under the CA1 pyramidal layer and the hippocampal fissure (hif). Leptomeninges, leptomeningeal blood vessels, and cortex blood vessels stained dark brown indicate Aβ accumulation (clear arrows). APP21 and WT rats did not have Aβ deposits in their hippocampus, brain cortex, or blood vessel walls.

**Fig 3:**
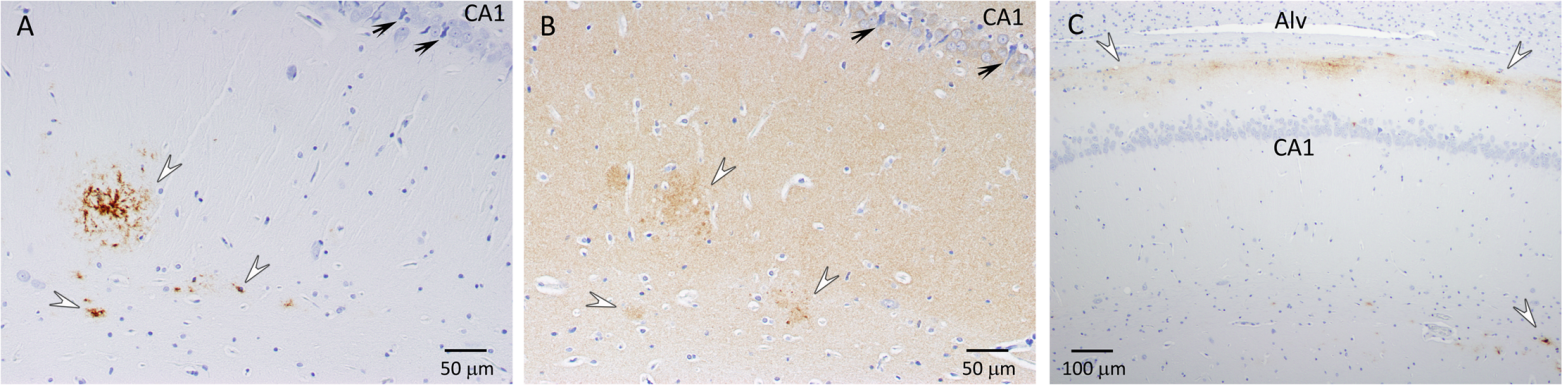
Amyloid-β plaques in the hippocampus of APP+PS1 rats. (A) Immunohistochemistry using 6E10 antibody against Aβ showing various sizes of dense Aβ plaques (white arrow heads); (B) Adjacent brain section that was used for immunohistochemistry using an antibody against Tau showing dense Aβ plaques also contained Tau (arrow heads). Necrotic neurons that were stained dark blue were not Aβ or Tau positive (arrows); (C) The presence of Aβ plaques under the alveolus hippocampus (Alv) and hippocampal fissure (arrow heads).

Immunohistochemistry results showed that a large area in between the CA1 pyramidal layer and the alveus of the hippocampus (Alv), as well as, the subiculum contained mostly diffuse and some dense Aβ plaques in all of the APP+PS1 rats (Fig 2A). In addition to sporadic dense Aβ plaques in the brain cortex, more frequent and dense Aβ plaques were present in between the granular layer of the DG and hippocampal fissure (hif; Fig 2A) in all of the APP+PS1 rats. Hippocampal dense and diffuse Aβ plaque area was determined by Image J and percent plaque area was calculated by dividing the area of the Aβ plaques by the total area of the hippocampus. Diffuse Aβ plaques occupied between 0.02% and 2% of the hippocampal area and averaged 0.8 ± 0.2% (mean ± SEM) in the APP+PS1 rats. Dense Aβ plaques occupied between 0.007% and 0.04% of the hippocampal area and averaged 0.2 ± 0.07% (mean ± SEM) in the APP+PS1 rats. Although WT and APP21 rats did not have any Aβ plaques, statistical analysis only yielded a trend for diffuse and dense plaque area of APP+PS1 rats compared to APP21 and WT rats (*F*_(2,10)_ = 3.15, *P* = 0.09 and *F*_(2,10)_ = 3.26, *P* = 0.08, respectively). This may have been due to the large variation in Aβ plaque formation in APP+PS1 rats. The Aβ plaques under the hif and in the brain cortex were dense and were very dark brown compared to the mainly diffuse plaques under the Alv. In addition, the dense plaques also reacted with the tau antibody (Fig 3). Although the diffuse Aβ plaques under the Alv occupied a very large area, they did not react with the tau antibody. Contrary to the APP+PS1 rats, the WT and APP21 rats did not show evidence of any Aβ plaque formation in the hippocampus or brain cortex. All WT, APP21, APP+PS1 rats showed some Aβ staining in their blood vessel content but there were no differences in Aβ positive hippocampal blood vessel area among the groups (*F*_(2,10)_ = 0.03, *P* = 0.97).

### Vascular changes

**Fig 4:**
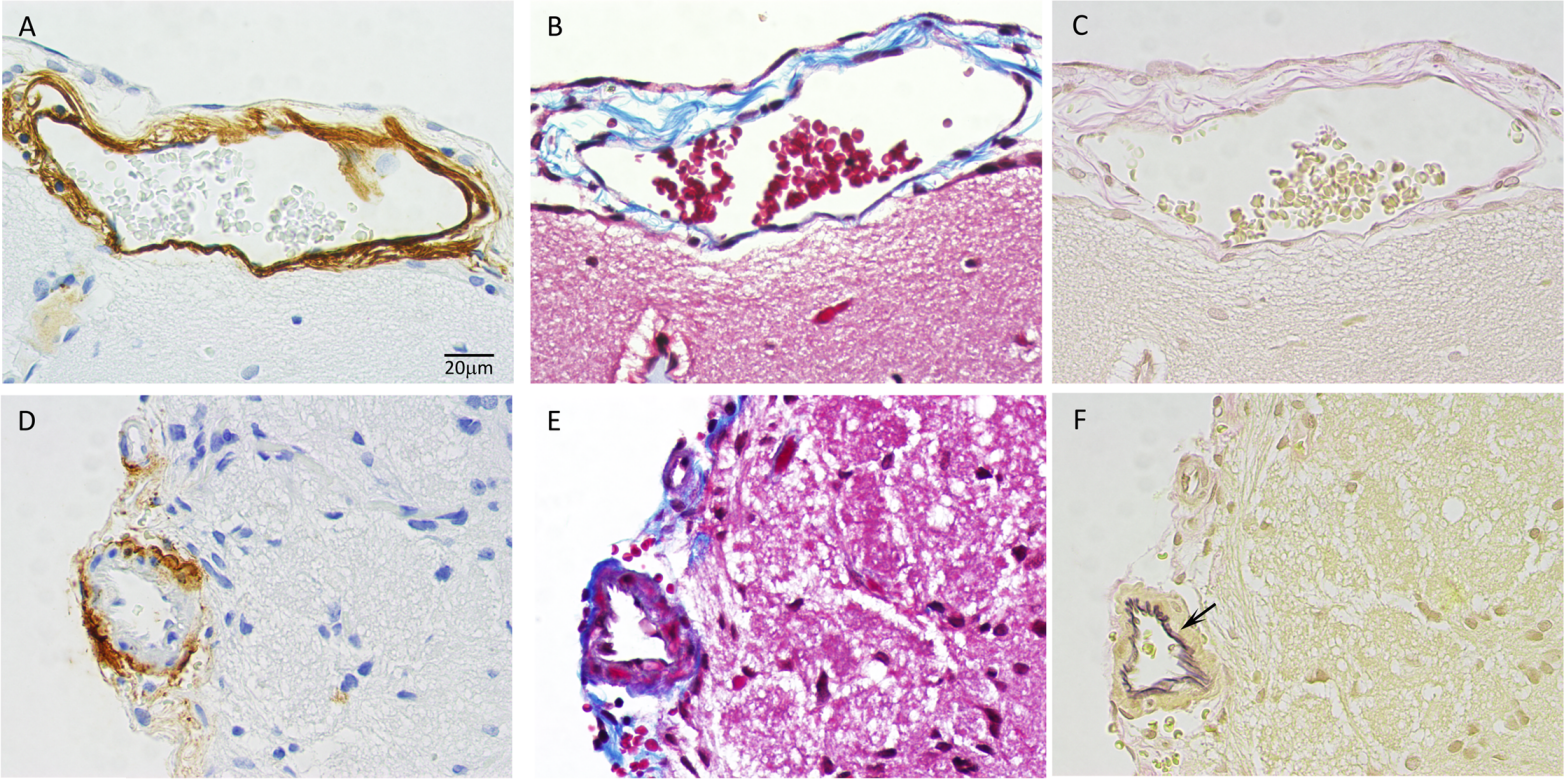
APP+PS1 rat leptomeningeal blood vessel walls contained Aβ deposits. Panels A, B and C show adjacent serial sections of an enlarged leptomeningeal vein and D, E and F show adjacent serial sections of a leptomeningeal artery. (A) Immunohistochemical staining for Aβ showing severe Aβ deposition in all layers of a leptomeningeal vein; (B) Trichrome staining resulted in a blue color in all layers of a vein indicating the presence of collagen related to the amyloid deposits; (C) Elastin staining showing minimal elastic fibers in veins; (D) Adventia and media of a leptomeningeal artery with strong Aβ staining and no Aβ staining of intima. (E) Trichrome staining resulted in a blue color in the adventia and media of an artery corresponding to Aβ deposits; (F) Elastin staining of a leptomeningeal artery with elastic fibers in intima (arrow).

**Fig 5:**
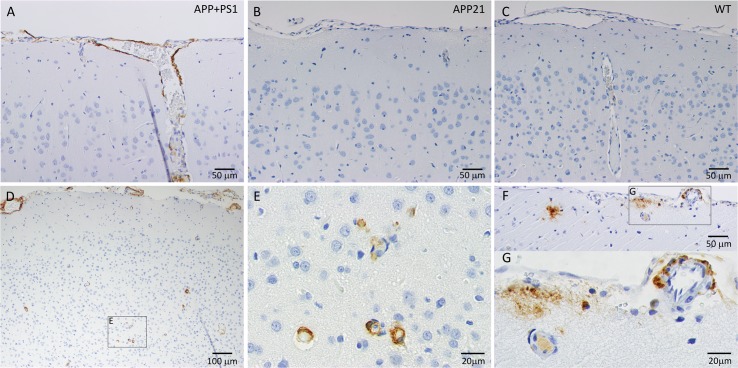
Immunohistochemical staining for Aβ of leptomeninges, leptomeningeal, and cortical blood vessels. (A) Extensive Aβ deposition in leptomeninges and leptomeningeal blood vessels and their distal branches; APP21 (B) and WT (C) rats did not have Aβ deposits in leptomeningeal or cortical blood vessels. (D, E, F and G) APP+PS1 cortex with cortical and leptomeningeal blood vessels with Aβ deposition; (E) Higher power image of cortical capillaries with Aβ deposits; (F and G) Leptomeningeal artery with Aβ deposits, dense and diffuse cortical Aβ plaques, and smaller cortical artery with Aβ positive content.

CAA, which is characterized by Aβ deposition in and around the blood vessel walls, was observed in all the APP+PS1 rats. The APP+PS1 rats had Aβ deposits in the leptomeninges and in the walls of leptomeningeal and cortical arteries and veins (Figs 4 and 5). Amyloid-β accumulated in all layers of the leptomeningeal vein walls (Fig 4A). In contrast, mainly the tunica adventia and occasionally the tunica media of the leptomeningeal arteries were affected with Aβ deposits (Fig 4D). Arteries and veins were distinguished by more pronounced staining of elastin in intima of arteries (Fig 4C and 4F). Fig 4 also shows that blood vessel walls with Aβ deposits were stained blue in trichrome staining, which indicates the presence of collagen. Amyloid-β deposition was observed in blood vessels of all sizes in the cortex, including capillaries, and there was no indication of inflammation around these blood vessels (Fig 5). The APP21 and WT rats did not have Aβ deposition in the blood vessel walls, however, the content inside the blood vessels showed a presence of Aβ, which could be explained by the presence of Aβ40 and Aβ42 in the serum of APP and APP+PS1 rats (Fig 5B, 5C and 5F).

**Fig 6:**
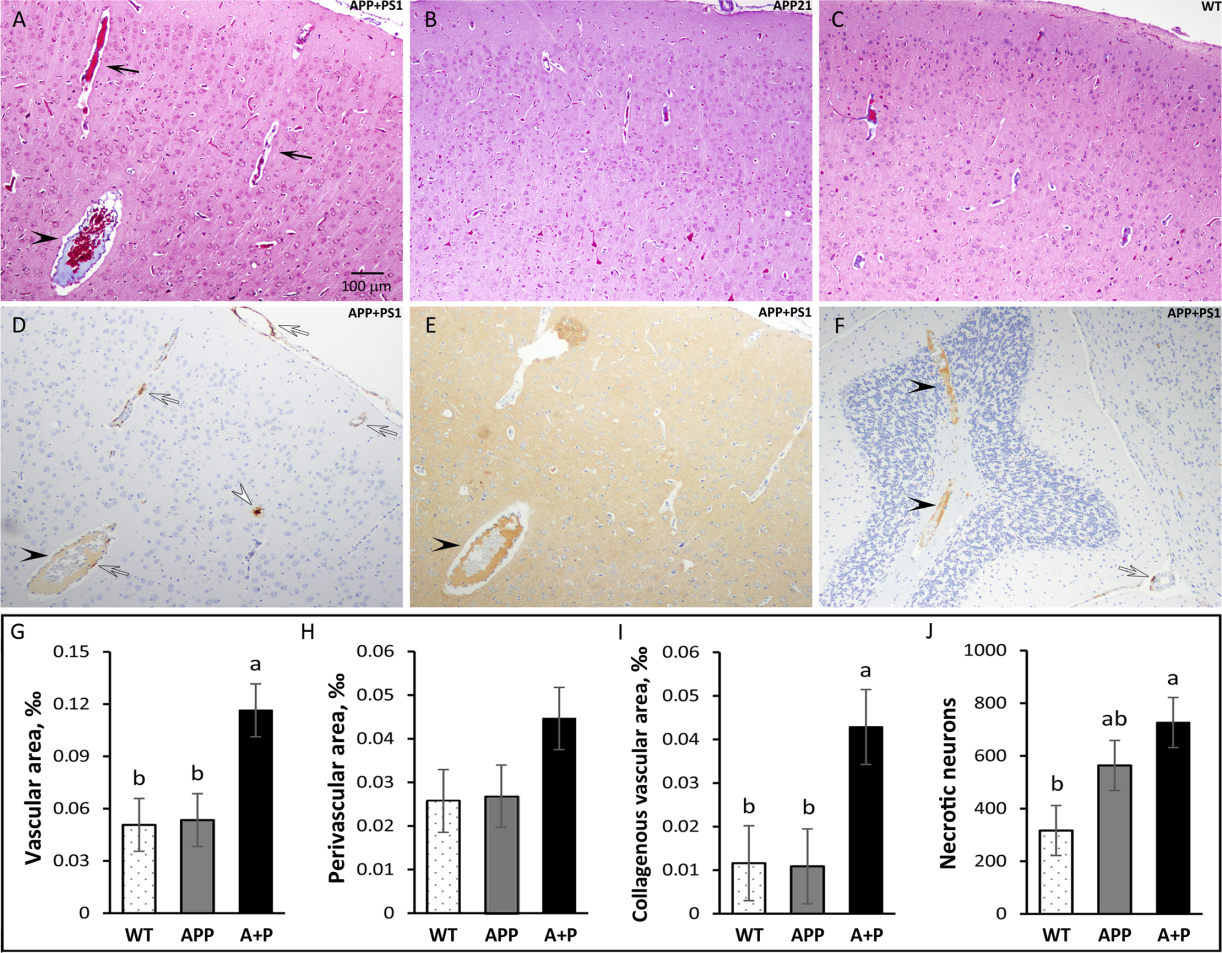
Vascular changes in APP+PS1 rats. Trichrome staining of the frontal cortex from APP+PS1, APP21, and WT rats showing enlarged blood vessels and enlarged perivascular area (black arrows) in an APP+PS1 rat (A) compared with APP21 (B) and WT (C) rats. Immunohistochemistry of an adjacent section to panel A stained with Aβ (D) and tau (E) antibodies showing venous collagenous stenosis which also contains Aβ and tau (black arrow heads). Panel D shows an APP+PS1 rat with cortical Aβ plaque (white arrow head) and CAA in cortical and leptomeningeal blood vessels (clear arrows); (F) Venous collagenosis containing Aβ in the cerebellum of an APP+PS1 rat. Vascular (G), perivascular (H), and collagenous vascular (I) areas which were expressed as a percent of total frontal cortex area were greater in APP+PS1 rats. (J) APP+PS1 rats (A+P) had the largest number of necrotic neuros, followed by APP21 rats. Letters a and b indicate significant differences (*P*<0.05).

CAA in APP+PS1 rats was accompanied by perivascular edema, which is characterized by enlarged perivascular space. The frontal cortex blood vessel perivascular space was 1.7-fold greater in APP+PS1 rats compared to WT and APP21 rats (*F*_(2,15)_ = 2.2, *P* = 0.15; Fig 6H). Percent of blood vessel area was calculated by dividing blood vessel area by the total frontal cortex area and was significantly (*F*_(2,15)_ = 6.0, *P* = 0.01; Fig 6G) greater in APP+PS1 rats compared to APP21 and WT rats. Enlarged blood vessels were also present in the hippocampus and other sections of the brain cortex in the APP+PS1 rats. Some of the cortical, hippocampal, and cerebellar veins were enlarged and contained collagenous stenosis or occlusions. This venous collagenosis was stained blue in trichrome staining, indicating the presence of collagen (Fig 6A). In addition, they also reacted with Aβ and tau antibodies (Fig 6D and 6E). The APP+PS1 rats had significantly (*F*_(2,15)_ = 4.5, *P* = 0.03) more collagenous blood vessels in frontal cortex compared to APP21 and WT rats (Fig 6I). Hemorrhagic areas were also observed in the brains of the APP+PS1 rats.

### Necrotic changes

Both the APP+PS1 and APP21 rats had many necrotic neurons and granular cells in the hippocampus and in the cortex. They were shrunken and had an unstained perineuronal space. The necrotic neurons either did not have a visible nucleus or they contained a pyknotic nucleus. Some of the necrotic neurons also showed evidence of mineralization with punctate deposits of mineral deposits at the cell surface. The necrotic neurons stained bright red with trichrome staining and were stained dark blue with Aβ and tau IHC staining. The necrotic neurons did not contain Aβ or tau (Fig 3A and 3B). The number of necrotic cells in the prefrontal cortex was significantly (*F*_(2,15)_ = 4.72, *P* = 0.03) greater in the APP+PS1 rats compared with the WT rats. APP21 rats had fewer necrotic cells than APP+PS1 rats but had more than WT rats (Fig 6J). In addition, one APP+PS1 rat had severe spongiosis, which occupied a large area in the motor cortex and medial parietal cortex.

### Serum Aβ40 and Aβ42 levels

ELISA results showed that serum Aβ40 levels of APP+PS1 and APP21 rats were 155 and 136 pg/ml, respectively and these amounts were not statistically different from each other (*F*_(1,8)_ = 0.1, *P* = 0.8). Serum Aβ42 levels of APP+PS1 and APP21 rats were 82 and 14 pg/ml, respectively and these were statistically different (*F*_(1,8)_ = 3.6, *P* = 0.1). WT rats did not have any measurable levels of Aβ40 or Aβ42 in their serum.

## Discussion

An evaluation of error performance found that APP21 and APP+PS1 rats showed poor memory for the location of the escape box during acquisition, retention, and reversal in the Barnes maze compared with the WT rats. This result is consistent with many spatial learning findings using rodent models of AD [18, 19, 20, 21], including performance in the Barnes maze [9, 12, 13, 14, 15, 22, 23].

However, surprisingly, the WT animals had significantly longer latencies than APP+PS1 animals during acquisition training and had a tendency towards longer latencies during reversal training. This trend was unexpected because previous studies with these models arrived at the opposite conclusion and found that WT had the shortest latencies and APP+PS1 rats had the longest latencies [9]. The current finding may have stemmed from differential levels of motivation; it is possible that the WT animals were no longer sufficiently aversively stimulated by the Barnes maze environment and thus unmotivated to complete the maze. This explanation seems plausible given that the WT animals had longer latencies but did not produce many errors. Subsequent studies may consider adding additional aversive stimuli to the testing environment such as white noise to improve motivation in the WT rats. Although anxiety levels were not measured in this study, other studies have found that Tg animals tend to have higher levels of anxiety [18, 24, 25, 26] and thus, it is possible that APP21 and APP+PS1 rats continue to be motivated to escape the aversive nature of the Barnes maze even after many training trials, whereas the WT animals may learn that the maze environment is no longer fear-inducing after being exposed to it over many trials.

Although APP21 rats did not have any Aβ plaques and visible histological vascular abnormalities, they had more necrotic neurons compared to WT rats. Poor error performance of APP21 rats in the Barnes maze may be due to greater number of necrotic changes in the brain. When the number of necrotic cell between the APP21 and WT rats were compared, statistical analysis showed a significantly greater (*P*<0.05) number of necrotic cells in APP21 rats. The current findings suggest that over-expression of APP on its own may result in neuronal pathology and a learning deficit and that Aβ plaques and CAA may not be required for such behavioral impairment.

The brain samples were obtained 5 to 7 months after the behavioral studies in order to maximize the pathological findings. Pathological changes increase with age in both AD patients and model animals, but memory deficiency can be observed at earlier ages before obvious histopathological changes occur [27]. Only APP+PS1 rats showed evidence of Aβ plaques in their brains, which indicates that the PS1 transgene is required for Aβ plaque formation. The Aβ plaques observed in the APP+PS1 rats were similar to those observed in AD patients. The brain cortex and the hippocampus of these animals had both dense and diffuse Aβ plaques. The area under the Alv, above the pyramidal layer of CA1 region of the hippocampus, had extensive diffuse plaques. Additionally, the area under the hif, above the granular layer of the DG, contained dense plaques. The common link here is that both the Alv and hif are connected to the lateral ventricle. Diffusion and bulk flow between CSF and the periventricular brain tissue enables bidirectional exchange of materials [28]. Thus, the accumulation of Aβ plaques in regions that are in close proximity to the Alv and hif may be due to reduced CSF outflow and turnover [29], as well as reduced perivascular drainage of interstitial fluid (ISF) and solutes [4] with age. The location of hippocampal plaques in the APP+PS1 rats suggests a role of reduced Aβ diffusion from periventricular tissues to CSF. Diffuse plaques observed above the CA1 region in the APP+PS1 rats corresponded to the area of the hippocampus with greater APP expression [8]. The APP21 rats expressed APP in pyramidal neurons of the CA1 and subiculum regions, but not in CA2. A similar pattern of highly concentrated Aβ plaques above the CA1 and subiculum regions was also previously observed in triple-transgenic AD mice [30, 31].

The APP+PS1 rats had severe CAA. CAA is defined as deposition of Aβ in the vessel walls in leptomeningeal and cortical arteries, arterioles, capillaries, and rarely veins, and is strongly associated with AD [3, 4]. CAA leads the tunica media to thicken or thin and degenerate, the arterial lumen to stenos or dilate, and corticomeningeal arterial vessel walls to dissociate. In CAA, smooth muscle cells are lost from artery walls and replaced by amyloid [32]. The leptomeningeal arteries and veins of APP+PS1 rats were severely affected with Aβ deposits. Additionally, the walls of the cortical arteries, veins, and capillaries also had Aβ deposits and APP+PS1 rats displayed strong Aβ staining in the media and adventia of the arteries. Similarly, CAA affects blood vessel media and adventia in AD patients [33, 34]. There are several hypotheses about the origin of Aβ in blood vessel walls: Amyloid-β in blood vessels walls may originate from circulation, smooth muscle cells, and pericytes within the vessel walls, or the neuropil in the course of perivascular drainage [3]. Age-dependent reduction in the perivascular drainage of ISF and solutes also contribute to CAA [4], which could explain the accumulation of Aβ in the veins of APP+PS1 rats. This corresponds to previous findings as Aβ deposition in cerebral veins has also been observed in other AD rat models [35]. The intimal layer of the leptomeningeal arteries of the APP+PS1 rats did not show any amyloid deposition and Aβ deposition appeared to begin in the adventia. These observations indicate that amyloid deposition may be originating from the ISF surrounding the blood vessels. Amyloid-β accumulation around the ISF drainage pathways along the perivascular channels around capillaries and arteries has also been previously identified in humans and animal models [36, 37]. The glympatic Aβ clearance model proposed by Peng and colleagues [38] suggests that Aβ clearance occurs through the ISF along a vein’s perivascular spaces. This model corresponds well with the CAA pathology observed in the APP+PS1 rats in this study. The only major difference between the CAA described in other AD model animals and the CAA in the APP+PS1 rats in this study is the presence of extensive Aβ deposits in the leptomeningeal vein walls.

Venous collagenosis, another vessel anomaly, was also detected in the APP+PS1 rats. Some of the APP+PS1 rats had enlarged cortical veins, enlarged perivascular space, and concentric stenosis containing collagen, Aβ, and tau. The increase in the size of the cortical blood vessel area and perivascular space in the APP+PS1 rats is similar to the increase in the perivascular space observed in AD patients [39]. Gao and colleagues [40] showed that patients with AD had periventricular hyperintensities and venulopathy and suggested that collagenosis causes dilation of the veins, venous insufficiency, vessel leakage, and vasogenic edema. Venous collagenosis manifests itself as noninflammatory, periventricular venulopathy with concentric collagen deposition and causes intramural thickening and stenosis, followed by luminal occlusion [41]. Increased vascular resistance and vasogenic edema compromise ISF circulation. This could reduce perivascular Aβ clearance pathways in AD and increase amyloid deposition. The prevalence of periventricular venous collagenosis increases with age and previous studies have found that approximately 65 percent of brain samples from patients over the age of 60 had periventricular venous collagenosis and spongiosis [42].

## Conclusions

The APP+PS1 rats showed many of the behavior and pathologic similarities to patients suffering from AD including learning and memory impairment, Aβ plaques in the cortex and hippocampus, and CAA in leptomeningeal and cortical blood vessels. The results of this study show that although APP21 animals had fewer histopathological abnormalities in their brains, they showed learning deficiency as signified by the Barnes maze acquisition and reversal results. This indicates that over-expression of APP may be sufficient to cause learning deficiency possibly caused by an increased number of necrotic neurons and that Aβ plaques and CAA are not required for learning impairment. The APP+PS1 rats showed a more severe memory and learning deficit and more pathological changes including AB plaques, CAA, and necrotic neurons resembling AD. Thus, CAA and Aβ plaques may exert an additional burden to cognitive processes and further reduce learning and memory.

## Supporting information

S1 Table Barnes maze data that was used for statistical analysis. (XLSX)

S2 Table Serum ELISA for Aβ40 and Aβ42, hippocampal amyloid beta plaque area and frontal cortex vascular area data that was used for statistical analysis. (XLSX)
